# Phytol-Loaded Solid Lipid Nanoparticles as a Novel Anticandidal Nanobiotechnological Approach

**DOI:** 10.3390/pharmaceutics12090871

**Published:** 2020-09-13

**Authors:** Tábata L. C. Lima, Luanda B. F. C. Souza, Lannya C. S. Tavares-Pessoa, Alaine M. dos Santos-Silva, Rômulo S. Cavalcante, Raimundo F. de Araújo-Júnior, Alianda M. Cornélio, Matheus F. Fernandes-Pedrosa, Guilherme Maranhão Chaves, Arnóbio Antônio da Silva-Júnior

**Affiliations:** 1 Laboratory of Pharmaceutical Technology and Biotechnology, Department of Pharmacy, Federal University of Rio Grande do Norte (UFRN), Gal. Gustavo Cordeiro de Farias, S/N, Petrópolis, Natal-RN 59072-570, Brazil; tabata.cunhalima@gmail.com (T.L.C.L.); claratavares@ufrn.edu.br (L.C.S.T.-P.); alaine.maria@hotmail.com (A.M.d.S.-S.); mffpedrosa@gmail.com (M.F.F.-P.); 2 Department of Clinical and Toxicological Analyses, Federal University of Rio Grande do Norte (UFRN), Gal, Gustavo Cordeiro de Faria, S/N, Petrópolis, Natal-RN 59084-100, Brazil; luandacanario@ufrn.edu.br; 3 Department of Morphology, Federal University of Rio Grande do Norte (UFRN), Natal-RN 59.078-970, Brazil; romulo.s.cavalcante@gmail.com (R.S.C.); araujojr.morfologia@gmail.com (R.F.d.A.-J.); aliandamaira@gmail.com (A.M.C.)

**Keywords:** solid lipid nanoparticles, phytol, *Candida* spp., 1,3-distearyl-2-oleyl-glycerol (TG1), anticandidal activity

## Abstract

Phytol is a diterpene alcohol and can be found as a product of the metabolism of chlorophyll in plants. This compound has been explored as a potential antimicrobial agent, but it is insoluble in water. In this study, we describe a novel approach for an interesting anticandidal drug delivery system containing phytol. Different formulations of phytol-loaded solid lipid nanoparticles (SLN) were designed and tested using a natural lipid, 1,3-distearyl-2-oleyl-glycerol (TG1). Different compositions were considered to obtain three formulations with 1:10, 1:5, and 1:3 *w/w* phytol/TG1 ratios. All the formulations were prepared by emulsification solvent evaporation method and had their physicochemical properties assessed. The biocompatibility assay was performed in the HEK-293 cell line and the antifungal efficacy was demonstrated in different strains of *Candida* ssp., including different clinical isolates. Spherical and uniform SLN (<300 nm, PdI < 0.2) with phytol-loading efficiency >65% were achieved. Phytol-loaded SLN showed a dose-dependent cytotoxic effect in the HEK-293 cell line. The three tested formulations of phytol-loaded SLN considerably enhanced the minimal inhibitory concentration of phytol against 15 strains of *Candida* spp. Considering the clinical isolates, the formulations containing the highest phytol/TG1 ratios showed MICs at 100%. Thus, the feasibility and potential of phytol-loaded SLN was demonstrated in vitro, being a promising nanocarrier for phytol delivery from an anticandidal approach.

## 1. Introduction

In the last few decades it is estimated that more than 150 million individuals develop severe fungal diseases every year, resulting in high rates of morbidity and mortality [1]. Yeasts belonging to the *Candida* genus are outstanding among opportunistic fungi. *Candida* species belong to the normal human microbiota and typically grow on the skin and mucosal surfaces such as the gastrointestinal genitourinary and respiratory tract [2,3]. Nevertheless, under favorable conditions, this opportunistic microorganism can rapidly change from commensal to pathogenic condition, causing a diverse spectrum of clinical diseases ranging from superficial and mucosal infections to invasive diseases associated with candidemia and metastatic organ involvement [4,5,6].

Vulvovaginal candidiasis (VVC) is a long-term condition that can severely affect the quality of life of affected women and is associated with considerable stress. VVC has been considered a global public health problem, affecting millions of women of all social strata. It causes great discomfort, impairs sexual relationships, and damages work performance [7]. Invasive infections due to *Candida* spp. are widely recognized as a major cause of morbidity and mortality in the healthcare environment and it is estimated that over 750,000 cases of invasive candidiasis are reported per year. [1,8]. In addition, approximately 40% of attributable mortality is found for patients with invasive candidiasis, despite therapy with conventional antifungal drugs [9,10]. At least 15 distinct *Candida* species may cause human diseases, whereas more than 95% of invasive infections are caused by the 5 most common *Candida* spp.: *Candida albicans*, *Candida glabrata*, *Candida tropicalis*, *Candida parapsilosis*, and *Candida krusei* [11].

For the treatment of different clinical forms of candidiasis, four major classes of antifungal agents have been widely used: the polyenes (i.e., amphotericin B and nystatin), the azoles (i.e., fluconazole, itraconazole, and voriconazole), the echinocandins (i.e., caspofungin, micafungin, and anidulafugin), and 5-flucytosine. However, the recent emergence of multidrug-resistant *Candida* species, such as *Candida auris*, has led to limitations to choose the appropriate antifungal therapy [12,13], which highlights the need for the discovery of new targets to treat both superficial and invasive candidiasis. In this context, plants and their natural compounds are excellent options to obtain a wide variety of drugs [14]. In this sense, it is possible to find reports in the literature showing that recently the antimicrobial activity of natural products has been widely researched and suggested as a possible therapeutic alternative [15,16,17].

Among natural products, essential oils have attracted attention because they are secondary metabolites that represent a very complex natural mixture with several components present in significantly different concentrations found in plants [18]. Diterpenes are a class of compounds commonly found in essential oils. These compounds are increasingly catching the attention due to their important biological activities [18,19,20]. Phytol(3,7,11,15-tetramethylhexadec-2-EN-1-OL), a branched long chain unsaturated diterpene member found abundantly in nature, is a product of the metabolism of chlorophyll in plants [21,22]. In the literature it was found that phytol exhibits a wide range of bioactivities including antioxidant [23,24], apoptosis-inducing and anti-angiogenic [25], and antimicrobial/antifungal activities [22,26]. Despite this good biological activity, phytol presents limitations as its solubility in aqueous solution, such as biological fluids, due to being a highly hydrophobic compound [27]. These characteristics lead to low bioavailability of this molecule. However, this problem can to be overcome with applications of effective drug delivery systems (DDSs) such as biocompatible/biodegradable nanocarriers [28].

In the literature, there are few studies that evaluate the advantages of drug delivery systems containing phytol. In previous studies, Sathya et al. (2020) [29,30,31] evaluated the activity of phytol-loaded poly (lactic-co-glycolic acid) nanoparticles as a possible treatment for Alzheimer’s disease. In addition, studies conducted by Islam et al. (2017) evaluated the antioxidant, toxicity, and cytotoxic effect of phytol-loaded nanoemulsions [24,32]. These studies suggests that phytol induces apoptosis dur to perturb the ionic balance, triggering changes in the membrane potential. The reported necrosis was associated with the augmented production of reactive oxygen cell species (ROSs). Studies performed by Sakthivel, Malar, and Devi corroborated with the apoptotic activity of phytol in A549 cells by a similar mechanism to that described above [25]. Nevertheless, the mechanism by which terpenes work as antimicrobial agent against bacteria and fungi has not yet been fully described. However, advanced studies suggest that it can act by inactivating enzymes and proteins important for the metabolism of microorganisms, as well as contributing to cell membrane damage due to intracellular potassium ion loss [33,34]. Thus, we hypothesized that this biological activity can be efficiently applied as a promising anticandidal agent, and its encapsulation in nanocarriers can improve this activity.

To date, no study has reported the evaluation drug delivery systems containing phytol as antimicrobial agents. Therefore, this fact makes this study a pioneer when it comes to the performance of phytol incorporated in nanocarriers, especially in the case of anticandidal activity.

Nanocarriers can be formulated using organic materials, playing a role as an important strategy for protecting active pharmaceutical ingredients. In addition, they change some properties of these drugs, such as the solubility, stability pharmacokinetics, biodistribution, and toxicity. Furthermore, the controlled drug release and site-specific drug delivery maximizes the clinical benefits while limiting their side effects [33,34,35]. In this background, solid lipid nanoparticles (SLN) are colloidal systems that have been explored to different purposes. On the contrary to nanoemulsions, SLN can be incorporated in ointments and creams as the final galenic dosage forms preserving the nanostructure. The dispersion of polymeric nanoparticles in these pharmaceutical dosage forms can also be a limitation.

The many advantages of SLN include good biocompatibility and low toxicity due to the lipid-like composition, as well as the sustained release for lipophilic drugs in an aqueous environment [36,37]. However, interaction of nanocarriers with cell membranes is a determinant factor for their cellular uptake, which could depend on nanoparticles size, shape, charge, and their surface characteristics [38]. In this context, it is important to evaluate the interactions of nanocarrier with targeted cells for improving the drug uptake. In this study, phytol was incorporated in SLN using different lipid/phytol ratios as a tentative to achieve a novel and promising nanobiotechnological approach with improved anticandidal activity, mainly considering the classical drugs.

## 2. Materials and Methods

### 2.1. Materials

The 1,3-distearyl-2-oleyl-glycerol (TG1) was donated by the Federal University of Piauí (Teresina, Brazil). The use of plant material was conducted under authorization from the National System for Management of Genetic Heritage and Associated Traditional Knowledge (SISGEN) no. ACB9C9D. Phytol diterpene was supplied by Sigma-Aldrich (São Paulo, Brazil). The polyvinyl alcohol (PVA), having a viscosity molecular mass of 4.7 × 10^4^ g/mol, was obtained from Vetec (São Paulo, Brazil). Dimethylsulfoxide (DMSO) and Alamar blue were purchased from Sigma-Aldrich (São Paulo, Brazil). The purified water (1.3 μS) was prepared from a reverse osmosis purification equipment model OS50 LX (Gehaka, São Paulo, Brazil). All other chemicals and reagents were of analytical grade.

### 2.2. Preparation of Nanoparticles

Solid lipid nanoparticles (SLN) were prepared by the emulsification solvent evaporation method [35]. For this, 1,3-distearyl-2-oleyl glycerol (TG1) was used as a lipid matrix. Briefly, the organic phase (OP) (6 mL) that was composed of dichloromethane (DCM) containing TG1 (0.5% *w/v*) and phytol (0.05% *w/v*, 0.1% *w/v*, and at 0.16% *w/v*) to obtain a TG1/PHY ratio of 1:10, 1:5, and 1:3 *w/w*, respectively. This organic phase was injected into the aqueous phase (AP) that was composed of 14 mL of the purified water containing the surfactant PVA (0.5% *w/v*) under magnetic stirring at 720 rpm at 25 °C ± 2 °C. The two OP and AP were previously filtered using 0.45 μm membranes (Sartorius, Goettingen, Germany). The emulsification was followed in Ultra-turrax equipment (IKA-Labortechnik, Staufen im Breisgau, Germany) at 20,000 rpm for 18 min and evaporation of the solvent occurred at 25 °C under magnetic stirring at 720 rpm overnight. Under same conditions, blank-solid lipid nanoparticles (SLN-B), without phytol, were prepared. The final colloidal dispersions were placed in hermetically sealed glass flasks and stored at 25 °C ± 2 °C. All experiments were performed in triplicate, and the data were expressed as mean ± standard deviation (SD).

### 2.3. Physicochemical Properties and Stability

#### 2.3.1. Particle Size and Zeta Potential Measurements

Mean particle size and polydispersity index (PdI) were assessed by using dynamic light scattering (DLS) in a particle size analyzer ZetaSize NanoZS (Malvern Instruments, Malvern, UK) at 659 nm wavelength, 173° detection angle, and at 25 °C. Zeta potential (ζ potential) measurements were performed in the same equipment through applying a field strength of about 5.9 V·cm^−1^ by using the electrophoretic mobility. The measurements were performed for at least 10 determinations for each sample diluted at 1:50 (*v/v*) with purified water. All experiments were performed in triplicate and data were expressed as mean ± standard deviation (SD).

#### 2.3.2. Attenuated Total Reflectance Fourier Transform Infrared (ATR-FTIR) Spectroscopy

The interactions of phytol with SLN were assessed using attenuated total reflectance Fourier transform infrared (ATR-FTIR) spectroscopy. The colloidal dispersions were lyophilized in a freeze-dryer (LIOTOP—L202) for 48 h. The spectra were recorded at 20 scans, with a resolution of 4 cm^−1^ between 4000 and 500 cm^−1^ for pure compounds (Phytol, PVA, TG1), SLN-B, and phytol-loaded SLN using three TG1/PHYTOL ratios (1:10, 1:5, and 1:3 *w/w*) using a FTIR-ATR spectrophotometer, SHIMADZU IR Prestige 21 (Tokyo, Japan).

#### 2.3.3. Physicochemical Stability

The colloidal dispersions of SLN were stored in the hermetically closed flasks at 25 °C for 6 weeks. At the intervals of 7 days, the samples were collected and had their size and zeta potential determined. The measurements were performed at 25 °C using the parameters described in Section 2.3.1. All analyses were performed in triplicate, and the data were expressed as mean ± standard deviation (SD).

#### 2.3.4. Atomic Force Microscopy (AFM) and Scanning Electron Microscopy (SEM)

The shape and surface of SLN-B and phytol-loaded solid lipid nanoparticles were observed by using AFM and SEM images. The dispersions were freshly diluted in purified water with a ratio of 1:25 (*v/v*) and dropped in a cover slip, dried under a desiccator with duration of 24 h, and then analyzed in an AFM and after in an SEM. The AFM analyses were performed using aSPM-9700 from Shimadzu (Tokyo, Japan) coupled with a cantilever non-contact, 1 Hz scanning. For the SEM images, one drop of each nanoparticle dispersion was placed on a washed microscope carbon slide and dried under a desiccator for 24 h and then in a field emission gun scanning electron microscope (FEG-SEM; Carl Zeiss, Auriga, East Lansing, MI, USA).

### 2.4. Drug-Loading Efficiency

Samples were centrifuged at 16,000× *g* for 60 min at 4 °C using the centrifugal filter (Merck Millipore, Darmstadt, Germany, Microcon, −10 kDa with Ultracel-10 membrane). After centrifugation, the filtered solution was analyzed to determine drug concentration using the UV–VIS spectrophotometry (Thermo Fisher Scientific, 60S Evolution, Madison, WI, USA) method at 239 nm, utilizing the equation from the fitted standard curve plot constructed previously [24]. The encapsulation efficiency (EE) was calculated by using Equation (1), where the total drug amount added (Drug_total_) and the non-entrapped drug (Drug_free_) in the filtered solution were considered [36].

(1) $$\mathrm{EE}\;\left(\%\right)=\frac{\left[\mathrm{Drug}\right]\mathrm{total}-\left[\mathrm{Drug}\right]\mathrm{free}}{\left[\mathrm{Drug}\right]\mathrm{total}}\times 100$$

### 2.5. Antifungal Activity

#### 2.5.1. *Candida* spp. Strains and Clinical Isolates

The following *Candida* reference strains obtained from the American Type Culture Collection (ATCC) and Centraalbureau voor Schimmelcultures (CBS) were used: *Candida albicans* ATCC 90028, *Candida dubliniensis* CBS 7987, *Candida tropicalis* ATCC 13803, *Candida parapsilosis* ATCC 22019, *Candida glabrata* ATCC 2001, *Candida rugosa* ATCC 10571, and *Candida krusei* ATCC 6258. Clinical isolates obtained from patients with vulvovaginal candidiasis (LMMM 92 and LMMM 100) or candidemia (LMMM 83, LMMM 85, LMMM 195, LMMM 249, LMMM 447, LMMM 704) used during this study belongs to the culture collection of the Medical and Molecular Mycology Laboratory, Clinical and Toxicological Analyses Department, at the Federal University of Rio Grande do Norte. This study was approved by the Local Research Ethics Committee (“Comitê de Ética em Pesquisa da Liga Norte Riograndense Contra o Câncer”) under the protocol number 042/042/2012. The strains were stored at −80 °C *in yeast extract* peptone dextrose (YPD) broth (10 g·L^−1^ yeast extract, 20 g·L^−1^ glucose, and 20 g·L^−1^ mycological peptone) containing 20% (*v/v*) glycerol and incubated in a rotator shaker (TE-420, Tecnal Piracicaba, Brazil) at 35 °C, 200 rpm, for 48 h for reactivation and verification of cell viability. Subsequently, 100 µL of cell suspensions was inoculated on the surface of Sabouraud Dextrose Agar (SDA; Oxoid, Basingstoke, Hampshire, UK) using a Drigalsky loop, and incubated for 48 h at 35 °C previously to antifungal susceptibility testing experimentation [38].

#### 2.5.2. Fungal Minimal Inhibitory Concentration (MIC) Determination of Phytol and Nanoparticles

For this study, seven different references and eight clinical strains of *Candida* spp. were tested. The antifungal susceptibility testing was assessed according to the Clinical and Laboratory Standard Institute (CLSI) protocol M27-A2 [39] (Wayne, PA, USA) with adaptations for natural products. For Minimal Inhibitory Concentration assay (MIC), the inoculum of all strains tested was obtained from 48 h cultivation on SDA at 35 °C and subsequently an initial suspension was prepared according to the McFarland scale 0.5 standard (1 to 5 × 10^6^ cells) by absorbance measurement using a UV–VIS spectrophotometer (Biochrom, Libra S32, Cambourne, UK). Then, two serial dilutions were made, firstly in saline solution (1:50) and then in Mueller–Hinton growth medium (HiMedia, Mumbai, India) (1:20). Subsequently, 100 μL aliquots of the final inoculum solution of each species were dispensed in different microtiter plates of 96 wells. Posteriorly, the strains were incubated, independently, with 100 μL of phytol (10,000–19.53 μg/mL), SLN-B (173.5–0.33 μg/mL), 10-SLN-PHY (62.5–0.12 μg/mL), 5-SLN-PHY (125–0.24 μg/mL), and 3-SLN-PHY (208.33–0.40 μg/mL). The samples were diluted in Mueller–Hinton growth medium. As a control for antifungal activity, fluconazole was used with concentrations ranging from 64 to 0.125 μg/mL. Finally, the plates were incubated at 37 °C and a test reading was taken after 48 h incubation. The MIC was considered the lowest concentration of the phytol and nanoparticles capable of inhibiting 50% or 100% of the growth of each strain, taking as reference the respective positive control (treated in the same manner, but without the treatment added to yeast cells).

### 2.6. Cell Viability Experiments

#### 2.6.1. Cell Culture

The human embryonic kidney epithelial cells lines (HEK-239) were maintained according to the specifications of the Cell Bank of Rio de Janeiro, Brazil. Briefly, the cells were maintained at 37 °C and 5% CO_2_ and cultured with DMEM (Dulbecco’s modified Eagle’s medium) culture medium supplemented with 10% of fetal bovine serum and 0.15% penicillin/streptomycin (10,000 U/mL) in 75 cm^2^ culture bottles.

#### 2.6.2. In Vitro Cell Viability Assay

The cell viability assay was determined by the resazurin reduction method, which evaluates cell viability by the enzymatic activity of mitochondrial dehydrogenases. For this, HEK-239 cells were grown in 96-well plates at a density of 4 × 10^3^ cells per well for 24 h until complete adhesion. After this period, cells were incubated for 24 and 48 h in the presence of different serial dilutions of samples. The SLN-B (0; 5.4; 10.8; 21.6; 43.3; 86.7; 173.5; 374.0 μg/mL), the 5-SLN-PHY (0; 1.9; 3.9; 7.8; 15.6; 31.2; 62.5; 125 μg/mL), and the 3-SLN-PHY (0; 3.2; 6.5; 13; 26; 52; 104; 208.3 μg/mL) were tested. The samples were diluted in culture medium without fetal bovine serum. For phytol, a stock solution of 5 mg/mL containing 2% DMSO was prepared and then was diluted (0; 3.9; 7.8; 15.6; 31.2; 62.5; 125; 250 μg/mL) in culture medium without fetal bovine serum. After the treatment time, 10 μL of resazurin solution (0.04%) was added to each well and the plate was incubated again for 4 h. Posteriorly, the absorbance was then measured on a microplate reader (Epoch, BioTek, Winooski, VT, USA) at wavelengths of 570 and 600 nm. The cell viability was calculated using the equation below (Equation (2)).

(2) $$\mathrm{Reduction}\;\mathrm{of}\;\mathrm{resazurin}\;\left(\%\right)=\frac{A570-\left(A600\times \mathrm{FC}\right)}{\mathrm{Ac}570-\left(\mathrm{Ac}600\times \mathrm{FC}\right)}\times 100$$

where A570 is the absorbance determined at 570 nm for samples, A600 is the absorbance determined at 600 nm for samples, Ac570 is the absorbance determined at 570 nm of the untreated group, Ac600 is the absorbance determined at 600 nm for the untreated group. The FC is the used correction factor.

For the determination of the cytotoxic concentration (CC_50_) values, we used nonlinear regression of concentration–response curves. The CC_50_ was defined as the concentration that reduced cell viability by 50% when compared to untreated controls [36]. All measurements were carried out in triplicate with three replicates for each dilution.

### 2.7. Statistical

All experimental values were expressed as mean ± standard deviation (SD). The pairwise comparisons of the analytical data were performed using the Student’s *t*-test. One-way analysis of variance (ANOVA) was applied for multiple comparisons, followed by the post hoc test of Dunnett’s vs. a control group. Data from cell viability assay were compared using one-way analysis of variance followed by Bonferroni’s test. A value of *p* < 0.05 was considered to be statistically significant.

## 3. Results and Discussion

### 3.1. Preparation of Drug-Loaded Solid Lipid Nanoparticles

SLN were successfully prepared using the selected parameters. Slightly turbid and opaque colloidal dispersions were observed 24 h after the preparation. Any phase separation was observed. The experimental design initially included the free-drug formulation (SLN-B) and three different phytol-loaded SLN using drug/TG1 ratios of 1:10 *w/w* (10-SLN-PHY), 1:5 *w/w* (5-SLN-PHY), and 1:3 *w/w* (3-SLN-PHY). All the formulations were prepared using the same experimental conditions. Table 1 shows the physicochemical properties of different formulations of SLN. The increasing amount of phytol in the SLN did not affect their mean diameter of approximately 300 nm, their particle size distribution (PdI < 0.2), or their negative zeta potential. In addition, high drug-loading efficiency was achieved for three formulations.

The preparation of monodisperse colloidal systems (PDI < 0.2) is important to predict their behavior and interaction with biological surfaces, mainly the capacity to overcome biological membranes [40,41]. Generally, the size of SLN ranges from 100 to 400 nm, yet negative zeta potential values between −20 and −40 mV were observed due to the nature of lipids used [42,43]. As already mentioned, phytol is a poorly water-soluble molecule. Anterior studies have reported encapsulation efficiency about of 90% for phytol in the PLGA nanoparticles [29,30,31]. This is an important parameter for characterizing SLN. Several factors such as the type and concentration of the lipid, as well as the used surfactant can affect this parameter for SLN. Certainly, changing these formulation parameters or nanoencapsulation method could enhance the entrapment of phytol in the SLN. However, the TG1 was chosen as the main and only structural lipid material due to be extracted from butter of *Platonia insignis*, a natural species that is part of the Brazilian biodiversity. PVA was used as surfactant due to its more predictable biocompatible formulations. The prepared SLN allowed its efficient dispersion in aqueous colloidal dispersions, with drug encapsulation efficiency being higher than 65% in the lipid matrix (Table 1). This was an important achievement, mainly considering the encapsulation efficiency of diterpenes in SLN [44,45,46]. Moreover, the SLN formulations containing phytol showed slightly lower pH, but no statistical difference was identified when compared to free-drug formulation. The physicochemical stability and morphology of SLN were also considered in this approach.

### 3.2. Morphology and Physicochemical Stability

**Figure 1:**
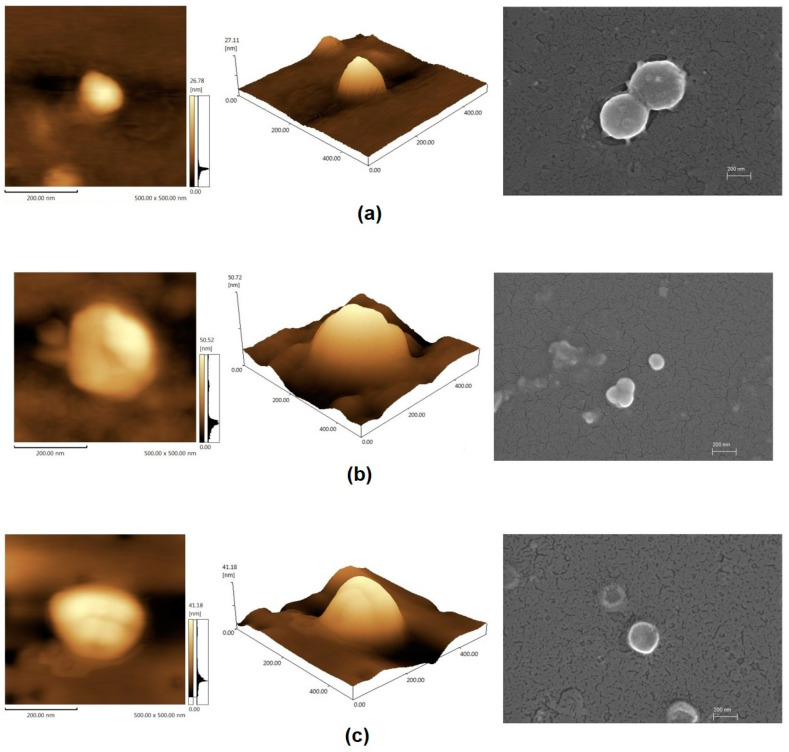
Morphology analyses of 2D and 3D atomic force microscopy (AFM) images and SEM images, respectively, of (**a**) blank-solid lipid nanoparticles (SLN-B), and phytol-loaded solid lipid nanoparticles (SLN-PHY) (**b**) 5-SLN-PHY and (**c**) 3-SLN-PHY.

**Figure 2:**
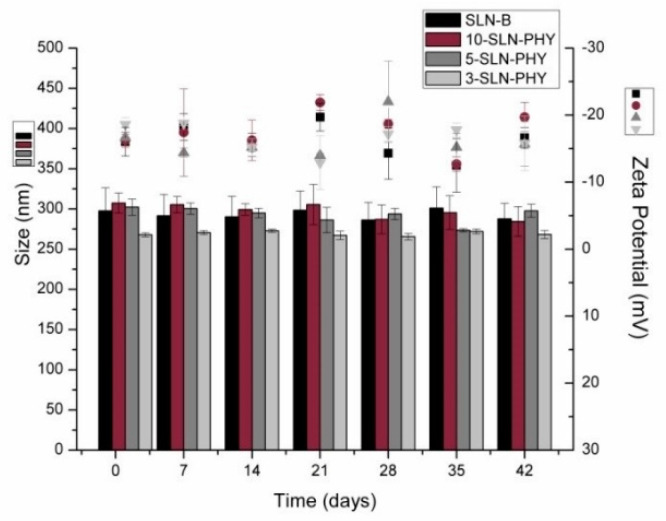
Mean diameter and zeta potential as a function of storage time for the solid lipid nanoparticles (SLN) and phytol-loaded solid lipid nanoparticles (SLN-PHY). Note: The data are expressed as mean ± standard deviation (SD) (*n* = 3).

Both AFM and SEM images have shown morphological and topographic aspects, such as shape and surface of SLN formulations. Different images of the formulation SLN-B (Figure 1a), 5-SLN-PHY (Figure 1b), and 3-SLN-PHY (Figure 1c) can be compared. The 2D and 3D AFM images can be seen at the left and center, while the SEM images can be observed at right of the pictures. Uniform and spherical solid lipid nanoparticles can be observed, with slightly smooth surfaces. This achievement corroborates with particle size experiments. In addition, the ability of different formulations for preserving the colloidal aspect and particle size was assessed for 6 weeks (Figure 2).

### 3.3. Mean Diameter and Zeta Potential as a Function of Storage Time for Different Formulations of SLN

The mean diameter, polydispersity index, and zeta potential were evaluated. The freshly produced samples were stored at room temperature (25 °C) and the first measurement was performed after 24 h, being repeated for every 7-day interval. This study was performed to assess the effect of drug loading on the physical stability of SLN. Different TG1/phytol ratios also were used. All the formulations remained stable, without significant changes in size and zeta potential. The phytol-loading at three different concentrations did not affect the physicochemical stability of SLN formulations. The same was observed for SLN-B (Figure 2). There were no significant variations between particle size and zeta potential values over the 6 weeks for SLN, being an indicative of absence of instability phenomena.

### 3.4. Attenuated Total Reflectance Fourier Transform Infrared (ATR-FTIR) Spectroscopy

**Figure 3:**
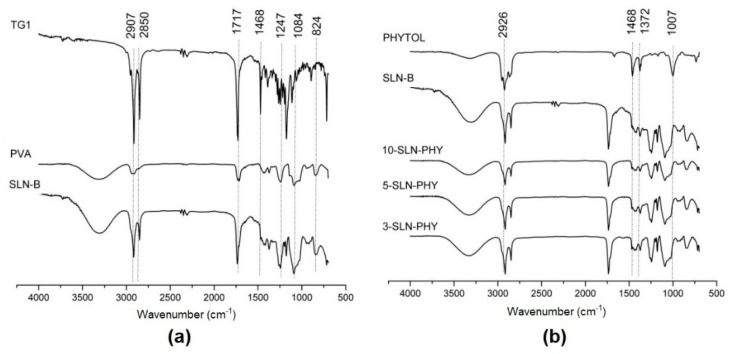
Attenuated total reflectance Fourier transform infrared (ATR-FTIR) spectra for compounds and different formulations of SLN. (**a**) Spectra for TG1, PVA, and blank-solid lipid nanoparticles (SLN-B); (**b**) spectra for phytol, SLN-B, and phytol-loaded nanoparticles (SLN-PHY).

The FTIR spectra recorded for TG1, PVA, and blank-solid lipid nanoparticles (SLN-B) are shown in Figure 3a. In the TG1 spectrum, it is possible to observe aliphatic C–H stretch of sp3 and sp2 carbons at 1926 cm^−1^ and 2850 cm^−1^, respectively. The bands recorded at 1750 cm^−1^ and 1468 cm^−1^ are assigned to C=O of ester and angular deformation of CH_2_. The C–O ester stretch and C–O of primary alcohol were recorded at 1247 cm^−1^ and 1048 cm^−1^, respectively. In the FTIR spectrum of SLN-B, the O–H recorded at 3250 cm^−1^ and bathochromic of C=O ester band to 1717 cm^−1^ occurred due to PVA covering on the surface of TG1 nanoparticles. Figure 3b shows FTIR spectra of pure phytol, SLN-B, and three formulations of drug-loaded SLN. The asymmetrical and symmetrical bending vibrations of C−H were recorded at 1468 cm^−1^ and 1372 cm^−1^, respectively, in the phytol spectrum. The C–O of primary alcohol was recorded at 1007 cm^−1^. Comparisons of three drug-loaded formulations suggested an augmented intensity of bands recorded at 1468 cm^−1^ and 1372 cm^−1^ and the bathochromic shift to lower wavenumber range, according the drug/TG1 ration enhanced in the SLN. The primary alcohol band of drug appeared to be overlapped in the nanoparticles.

FTIR spectra was applied as an important tool to characterize SLN. The suggested shifts identified in the FTIR spectra of SLN-B and drug-loaded SLN are common in self-assembled nanoparticles, in which their interactions determines their deposition in the particles [47,48]. The observed differences in the FIR spectra corroborates with drug-loading experiments. The shifts in spectra of formulations can be associated with drug-loading in the SLN. This achievement is so important to explain possible differences among the biological activity of different samples [31,49]. In this context, the experimental data support the expected ability of SLN for loading of poorly soluble diterpene drugs in an aqueous environment, which can be explored as a potential topical drug delivery system [50].

The use of TG1 as lipid matrix in the SLN formulations is due to this compound being extracted from butter of *Platonia insignis*, a natural species from Brazil. In studies performed by Feitosa et al. (2015) [51], biochemical and hematological parameters were evaluated, as well as histopathological analysis in brain areas of adult rats treated orally with the TG1 triglyceride. The authors concluded that treatment with TG1 did not produce hematological, biochemical, or histopathological alterations in rats, suggesting low toxicity. In addition to in vitro studies, in vivo studies have been carried out to prove that this compound is biocompatible. This fact is particularly important, mainly considering the mucosal environment affected by candidiasis and candidemia, in which an unprovable homogeneous dispersion of phytol drug is expected.

### 3.5. Anticandidal Assays

#### 3.5.1. Minimal Inhibitory Concentration (MIC) of Growth in Reference Strains of *Candida* spp.

In this approach, the anticandidal experiments assessed the MICs and minimum fungicidal concentration (MFC) with the established broth microdilution assay [38]. Thus, it is interesting to report that all MIC values reported in this section corresponded to the lowest dilutions tested for each sample. For this, the maximum concentration of free phytol (PHY) tested was 10,000 μg/mL (10 mg/mL), whereas the phytol concentration in the nanoparticles varied according to the drug proportion in the SLN formulations, reaching concentrations 40-fold lower than those for free phytol. Despite three drug-loaded SLN formulations having similar EE%, these three samples had different drug rates. We realize that the TG1/phytol ratio is an important variable that affects SLN efficacy as an antimicrobial agent.

At the concentrations tested, SLN-B was not able to inhibit fungal growth. Conversely, results from experiments using PHY and 10-SLN-PHY showed that both treatments were able to inhibit 50% of fungal growth for all the strains tested. The PHY presented MICs ranging from 2500 to 625 μg/mL, whereas the 10-SLN-PHY showed a more remarkable inhibitory activity, where MICs ranged from 15.62 to 1.95 μg/mL. For *C. albicans* ATCC 90028, *C. dubliniensis* CBS 7987, *C. parapsilosis* ATCC 22019, and *C. glabrata* ATCC 2001, the nanoparticles were able to improve the phytol antifungal activity by decreasing MICs of approximately 300 times. PHY and 10-SLN-PHY comparisons allow us to realize that *C. glabrata* ATCC 2001 was the most susceptible strain, with a 50% inhibition of visual growth at concentrations of 625 and 1.95 μg/mL for PHY and 10-SLN-PHY, respectively. The *C. krusei* ATCC 6258 showed the highest 50% growth MICs, with concentrations of 2500 and 15.62 μg/mL for PHY and 10-SLN-PHY, respectively. When we analyzed the results for the other strains, we verified that the SLN was able to lower the MIC obtained for PHY (10-SLN-PHY). This was observed for *C. albicans* ATCC 90028 and *C. dubliniensis* CBS 7987 (Table 2).

To achieve 100% of growth inhibition, we increased phytol concentration in the nanoparticle formulations. Subsequent tests were performed with the 5-SLN-PHY and 3-SLN-PHY and, as expected, the latest nanoparticles were able to induce a total visual growth inhibition of yeast cells at concentrations of 0.24 and 0.40 μg/mL, respectively.

Among the strains tested, it is interesting to observe that the most remarkable result was found for the *C. krusei* ATCC 6258 strain, an intrinsically fluconazole-resistant *Candida* species (MIC of 16 µg/mL) that showed very low MICs when grown in the presence of nanoparticles, with a concentration of 0.24 µg/mL. Following a similar trend, low MICs were also found for *C. tropicalis* ATCC 13803, *C. parapsilosis* ATCC 22019, and *C. glabrata* ATCC 2001 strains, where the MIC for 5-SLN-PHY (0.24 µg/mL) was lower than the one found when cells where grown in the presence of fluconazole (0.5 µg/mL). Results regarding inhibition of the other reference strains were also satisfactory, considering that MIC of nanoparticles is at least like what was found for fluconazole.

In order to verify if the visual inhibition of 100% of yeast cells growth was due to a fungicidal action of the nanoparticles, we streaked out aliquots of each well on the surface of SDA in order to verify the absence or presence of fungal growth. As there was yeast growth on SDA, except for the negative control (as expected), the nanoparticles may have had a rather fungistatic action on *Candida* spp. cells.

#### 3.5.2. Fungal Minimal Inhibitory Concentration (MIC) in *Candida* spp. Clinical Isolates

Under the same experimental conditions, PHY and SLN were tested against eight *Candida* spp. strains isolated from patients with vulvovaginal candidiasis and candidemia. The results are presented in Table 3. Again, the SLN-B was not able to inhibit yeast growth. However, contrary to the results found for the reference strains, the concentrations of PHY and 10-SLN-PHY tested were also not able to inhibit yeast growth of clinical isolates.

Interesting results were achieved after treatment with 5-SLN-PHY and 3-SLN-PHY as seven out of eight strains tested had a 100% MIC equivalent to 0.24 μg/mL and 0.40 μg/mL, respectively. It is important to mention that the MICs obtained with the use of these nanoparticles were lower than the ones found when fluconazole was used (except for the *C. albicans* LMMM 92 strain).

The MICs for 100% visual inhibition after treatment with 5-SLN-PHY were also considered an incredible achievement, mainly considering the results with fluconazole. The achieved concentration of 0.24 μg/mL for *C. parapsilosis* LMMM 83, *C. parapsilosis* LMMM 85, *C. glabrata* LMMM 704, and *C. krusei* LMMM 249 was lower that determined when the fluconazolewas used. Conversely, *C. albicans* LMMM 100 showed higher MIC (7.81 μg/mL), while *C. tropicalis* LMMM 195 and LMMM 447 growth was not inhibited, even at the highest concentration.

Fluconazole was used as a control antifungal drug that is commercially available and has previously known anticandidal activity. It is known that this drug is fungistatic and acts by inhibiting the biosynthesis pathway of ergosterol by impairing the activity of the enzyme lanosterol 14-α-sterol demethylase [52]. The results showed that the use of nanoparticles was able to produce a total visual growth inhibition at lower concentrations than fluconazole, for both reference and clinical isolates (Table 2 and Table 3). In fact, we must consider that the drug incorporated within SLN has advantages when compared to their unincorporated counterparts, such as a greater penetration into fungal cells, favored by the small size presented by the delivery system [50,53]. The same reason may justify the fact that 10-SLN-PHY has a MIC of 50% inhibition activity approximately 300 times lower when compared to the PHY in the assays containing *Candida* spp. reference strains. It was also shown that when the phytol concentration incorporated in the nanoparticles was increased from 10-SLN-PHY to 5-SLN-PHY or 3-SLN-PHY, this inhibition reached 100%. Other reported studies described nanotechnology approaches as strategy to obtain better results regarding antifungal activity [54,55,56].

Results from SLN tests with the *Candida* spp. reference strains showed that these nanoparticles were able to induce 100% growth inhibition. Here, two important aspects should be considered: the composition of the nanoparticles and their surface charge. The TG1, a constituent of the lipid matrix, is a triacyl glyceride of natural origin isolated from the plant *Platonia insignis* Mart [51]. There are few reports in the literature regarding its pharmacological properties. In addition to the cicatrizing activity cited below, the in vivo toxicity of this compound was also evaluated [57], and to date there are no reports of antifungal activity. Thus, we believe that through a synergistic activity between PHY and TG1 we are able to induce antifungal activity for the compound of natural origin [53].

For tests with clinical isolates, we showed that the tested concentrations of PHY, SLN-B, and 10-SLN-PHY were not effective in inducing growth inhibition. This finding may be related to possible antifungal selective pressure, with previous fungal cells exposure during patients’ treatment. Eventually, horizontal transmission of resistant strains may occur among hospitalized patients, making the issue of resistance to conventional antifungal treatments a very serious problem [58]. Nevertheless, the visual growth inhibition of the clinical isolates after treatment with the other nanoparticles was similar to what was observed for the reference strains. Among the formulations tested, the one with the best biological activity was 5-SLN-PHY, because it was able to inhibit seven out of eight clinical strains in non-cytotoxic concentrations (0.24 µg/mL of phytol; Table 3).

In this study, we were able to show that phytol was effective against several medically important *Candida* species. Among them, *C. albicans* is considered the most virulent and frequently isolated *Candida* spp. from different anatomical sources [59]. *C. albicans* has the high ability to adhere to human epithelial cells; produces several enzymes such as hemolysins, phospholipases, and proteinases; and is a strong biofilm producer [60,61].

In addition, *C. glabrata* was generally the most susceptible species to the phytol activity. This is the second most frequently isolated species from patients with candidemia in North America and Europe [60]. This species shows pathogenic potential due to its ability to lyse erythrocytes and produce phospholipases [61]. Although it is a haploid and asexual yeast, the various selective pressures routinely suffered eventually favor its genetic diversity and evolution, causing alterations in genes that regulate the expression of virulence factors, possibly helping greater host adaptations [62]. Miranda-Cadena et al. (2018) reported the isolation of *C. glabrata* strains that showed a simultaneous cross-resistance profile to different azoles (fluconazole, itraconazole, and miconazole) [63].

The biological activity found in the present study for *C. parapsilosis*, *C. tropicalis*, and *C. krusei* are also relevant since these non-*C. albicans Candida* species are emerging in cases of invasive candidiasis. In Brazil, *C. albicans*, *C. tropicalis*, *C. parapsilosis*, and *C. glabrata* are recognized as the main etiological agents of bloodstream infections [64]. Specifically referring to *C. parapsilosis*, it can be said that it is considered an emerging species related to invasive candidiasis [64], as well as VVC [65]. Hashemi et al. (2019) investigated species distribution and antifungal susceptibility of *Candida* spp. isolated from patients diagnosed with VVC and found that *C. parapsilosis* was the third most prevalent species, while *C. albicans* was the first [66].

In the literature, we have not found data on the precise mechanism of phytol’s antifungal activity, but as it is a component present in essential oils, we can suggest that it is causing yeast death due to a de-stabilization in the fungal cell membrane [67]. As a way of reinforcing this hypothesis, in the study developed by Bakkali and co-workers (2008), the authors postulated phytol-containing essential oils to destabilize eukaryotic cells, increasing cell membrane permeability, as well as promoting mitochondrial membrane depolarization [68]. In addition, Barros de Alencar and co-workers (2017) extensively reviewed the action of diterpenes against neglected diseases and reported that substances with one or more –OH active groups that also have antioxidant activity can increase membrane permeability of a variety of organisms such as bacteria, fungi, and viruses [69].

### 3.6. In Vitro Viability Assay

Considering the anticandidal assays, we performed cell viability assays for the two best formulations, the 5-SLN-PHY and 3-SLN-PHY samples. This experiment was performed for evaluating in vitro biocompatibility of free-drug SLN and understanding how the phytol changed this property. In addition, the cytotoxic effect of phytol was previously reported in the literature [24,25], and the possible enhancement of this biological effect reinforced the ability of the nanocarrier to improve the phytol interaction with cells.

**Figure 4:**
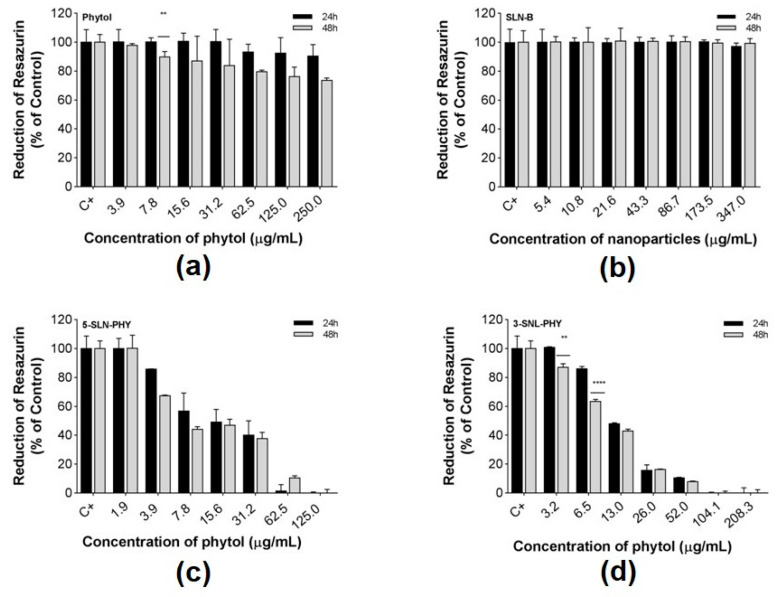
Cell viability in HEK-293. Assay performed after the cells incubation with (**a**) phytol; (**b**) blank-solid lipid nanoparticles (SLN-B); (**c**) phytol-loaded in solid lipid nanoparticles 1:5 (5-SLN-PHY); (**d**) phytol-loaded in solid lipid nanoparticles 1:3 (3-SLN-PHY). All measurements were carried out in triplicate with three replicates for each dilution. The cell viability was defined in comparison to untreated controls. The statistical was performed using the two-way ANOVA of distinct concentrations tested at 24 h vs 48 h. A *p* < 0.01 was represented as ** and *p* < 0.001 as ****.

The results of the cell viability assay are shown in Figure 4. After analyses, the results indicated that PHY at concentrations ranging from 250 to 3.9 μg/mL was well tolerated by HEK-293 cells, demonstrating a low cytotoxic effect over time and indicating cytotoxic concentration to 50% of the cells (CC_50_) was >250 μg/mL (Figure 4a). In the first 24 h, at all concentrations tested, cell viability was estimated to be equal to or greater than 90%. In results for 48 h, cell viability decreased, and a dose-dependent profile was identified. Concentrations equal to or lower than 62.5 μg/mL were considered biocompatible for the cells that presented more than 80% of cellular viability. In addition, the SLN-B was also well tolerated at the tested concentrations, even after 48h of incubation (Figure 4b).

Tests with 5-SLN-PHY showed the occurrence of a dose-dependent cytotoxic effect and the fact that within the first 24 h of incubation, the highest tested concentration (125 μg/mL) was toxic to cells (Figure 4c). This effect was enhanced when the drug loading was augmented in 3-SLN-PHY (Figure 4d). This fact can be better observed in Table 4. The estimated CC_50_ of 13.34 and 10.5 μg/mL were observed for 5-SLN-PHY and 3-SLN-PHY, respectively.

These achievements proved the expected ability of SLN for improving the drug uptake. Solid lipid nanoparticles are drug delivery systems that are capable of improving the bioavailability of poorly soluble molecules in aqueous environments, as well as promoting protection, high stability, and controlled release of the compound that is dissolved or dispersed in the lipid matrix [50]. Regarding cytotoxicity, we can say that it is very important to perform in vitro viability assays carried out in the initial stages of a study, since they allow access to preliminary data regarding a possible cytotoxicity of the new compound being tested [70].

As shown in Figure 4c,d, PHY-loaded SLNs caused a dose-dependent cytotoxic effect as a function of time at the highest concentrations tested. Islam and co-workers (2017) evaluated the cytotoxic potential of phytol in *Artemia salina* and suggested that the cytotoxicity of this compound is due to a succession of events, among them, failure of ATP supply, changes in membrane potential, probable increase in the formation of reactive oxygen species (ROS), and changes in Na^+^/K^+^ pump functioning, resulting in a greater influx of Na^+^ and Ca^2+^ as well as changes in membrane-selective permeability, which leads to cell death [32].

SLN-B was not cytotoxic to HEK-239 cells at the concentrations tested, suggesting that the system is biocompatible. Indeed, Mendes and co-works (2015) evaluated the healing potential of TG1 in vivo and found that pharmaceutical formulations containing up to 7.5 g of TG1 were not considered toxic and were effective in the process of reestablishing the integrity of the animals’ skin [57]. The experimental data discussed in this study corroborates the potential and promising nature of TG1-based SLN as a biocompatible and efficient nanocarrier for phytol. In addition, the ability of nanoparticles to facilitate the drug transport through the biological membranes is well explored [71]. An increased uptake of phytol supposedly increases its inhibitory effect, a relevant property mainly considering its antimicrobial use.

As previously mentioned, there are no reports in the literature regarding the elucidation of the mechanism of action of either free or incorporated phytol against *Candida* spp. However, it is known that molecules with antifungal activity when in contact with fungal cells first interact with the constituents of the cell wall, mainly the polysaccharides, and then with cell membrane composed of phospholipids and ergosterol. Finally, there is interactions with the other internal structures such as organelles [72,73]. Nevertheless, we currently did not aim to investigate the molecular interaction and mechanism of action of the nanoparticles with *Candida* cells. However, given their size, we assume that phytol nanoparticles are able to cross the fungal cell wall and interact with plasma membrane and other internal organelles, possibly interfering with structure organization and metabolic pathways that are important for fungal cell fitness, leading to K^+^ leakage [33,34]. Further analysis is needed to investigate the mechanism of action.

## 4. Conclusions

The results presented in this study explored the phytol as a bioactive compound that is able to be used and an innovative anticandidal compound incorporated in solid lipid nanoparticles. We realize that it is possible to modulate the anticandidal effect of formulations by hanging phytol/TG1 ratio. The experimental design was efficient to prove this purpose. The prepared formulations exhibited interesting properties such uniform particle size distribution, phytol content, and physical stability. The biocompatibility of free-drug SLN was assessed in HEK-3 cells. The experimental achievements demonstrated the potential of this nanocarrier, which was prepared using a naturally occurring lipid matrix and low surfactant concentrations. As we hypothesized, the drug-loaded SLN presented a dose-dependent anticandidal effect, and was able to inhibit the fungal proliferation of reference strains, as well as clinical isolates obtained from patients with vulvovaginal candidiasis or candidemia. These achievements can be considered excellent, mainly considering the superior performance of SLN formulations compared to fluconazole drug. The experimental data discussed in this study certainly support further experiments to stabilize these formulations as powder and in vivo studies in specific candidemia models of this promising nanobiotechnological approach for anticandidal therapy.

## 5. Patients

The clinical isolates used in this study were collected from patients with vulvovaginal candidiasis (LMMM 92 and LMMM 100) or candidemia (LMMM 83, LMMM 85, LMMM 195, LMMM 249, LMMM 447, LMMM 704). These belong to the culture collection of the Medical and Molecular Mycology Laboratory, Clinical and Toxicological Analyses Department at the Federal University of Rio Grande do Norte. This study was approved by the Local Research Ethics Committee (“Comitê de Ética em Pesquisa da Liga Norte Riograndense Contra o Câncer”) under the protocol number 042/042/2012.

## Figures and Tables

**Table 1:** Physicochemical properties of different solid lipid nanoparticles (SLN) formulations.

Nanoparticles	TG1/Phytol Ratio	pH	Size (nm)	PdI	ZP (mV)	EE (%)
SLN-B	-	6.8	297.6 ± 28.5	0.15 ± 0.03	−16.0 ± 2.2	-
10-SLN-PHY	1:10	6.4	307.4 ± 12.5	0.15 ± 0.07	−16.3 ± 11.1	67.38
5-SLN-PHY	1:5	6.5	302.2 ± 10.0	0.12 ± 0.03	−16.7 ± 0.5	68.74
3-SLN-PHY	1:3	6.5	297.9 ± 2.4	0.14 ± 0.02	−18.6 ± 1.0	68.20

Note: Data are expressed as mean ± standard deviation (*n* = 3). TG1, 1,3-distearoyl-2-oleoylglycerol; PdI, polydispersity index; ZP, zeta potential; EE%, encapsulation efficiency.

**Table 2:** Minimum inhibitory concentration values of phytol and SLN against *Candida* spp. reference strains determined with the broth microdilution assay.

*Candida* spp. Reference Strains	MIC (µg/mL) of Formulations					
	MIC 50				MIC 100	
	FLU	PHY	SLN-B	10-SLN-PHY	5-SLN-PHY	3-SLN-PHY
*C. albicans* ATCC 90028	0.125	2500	>173.5	7.81	0.24	0.40
*C. dubliniensis* CBS 7987	0.5	2500	>173.5	7.81	0.24	0.40
*C. tropicalis* ATCC 13803	0.5	1250	>173.5	7.81	0.24	0.40
*C. parapsilosis* ATCC 22019	0.5	1250	>173.5	3.90	0.24	0.40
*C. glabrata* ATCC 2001	0.5	625	>173.5	1.95	0.24	0.40
*C. rugosa* ATCC 10571	0.125	1250	>173.5	15.62	0.24	0.40
*C. krusei* ATCC 6258	16.0	2500	>173.5	15.62	0.24	0.40

Notes: FLU: fluconazole; ATCC: American Type Culture Collection; CBS: Centraalbureau voor Schimmelcultures; *C.*: *Candida*; MIC 50%: minimum inhibitory concentration that inhibited 50% growth; MIC 100%: minimum inhibitory concentration that inhibited any visible growth.

**Table 3:** Minimum inhibitory concentration values of phytol and SLN against *Candida* spp. clinical isolate strains determined with the broth microdilution assay.

*Candida* spp. Clinical Isolates	MIC (µg/mL) of Formulations					
	MIC 50				MIC 100	
	FLU	PHY	SLN-B	10-SLN-PHY	5-SLN-PHY	3-SLN-PHY
*C. albicans* LMMM 92	0.5	>10,000	>173.5	>62.5	>125	>208.3
*C. albicans* LMMM 100	0.5	>10,000	>173.5	>62.5	0.24	0.40
*C. tropicalis* LMMM 195	1.0	>10,000	>173.5	>62.5	0.24	0.40
*C. tropicalis* LMMM 447	2.0	>10,000	>173.5	>62.5	0.24	0.40
*C. parapsilosis* LMMM 83	0.125	>10,000	>173.5	>62.5	0.24	0.40
*C. parapsilosis* LMMM 85	16.0	>10,000	>173.5	>62.5	0.24	0.40
*C. glabrata* LMMM 704	2.0	>10,000	>173.5	>62.5	0.24	0.40
*C. krusei* LMMM 249	16.0	>10,000	>173.5	>62.5	0.24	0.40

Notes: FLU: fluconazole; LMMM: microbiology and medical mycology laboratory; *C.*: *Candida*; MIC 50%: minimum inhibitory concentration that inhibited 50% growth; MIC 100%: minimum inhibitory concentration that inhibited any visible growth.

**Table 4:** CC_50_ values for different samples.

Formulations	(CC50 µg/mL)
PHY	>250
SLN-B	>347
5-SLN-PHY	13.34
3-SLN-PHY	10.50

Notes: Data are expressed as mean ± standard deviation (SD) from three independent experiments, with each treatment performed in triplicate; CC_50_: concentration that was cytotoxic for 50% of the cells.
